# HDAC8 overexpression in mesenchymal stromal cells from JAK2^+^ myeloproliferative neoplasms: a new therapeutic target?

**DOI:** 10.18632/oncotarget.15969

**Published:** 2017-03-07

**Authors:** Teresa L. Ramos, Luis Ignacio Sánchez-Abarca, Alba Redondo, Ángel Hernández-Hernández, Antonio M. Almeida, Noemí Puig, Concepción Rodríguez, Rebeca Ortega, Silvia Preciado, Ana Rico, Sandra Muntión, José Ramón González Porras, Consuelo Del Cañizo, Fermín Sánchez-Guijo

**Affiliations:** ^1^ Universidad de Salamanca-IBSAL-Hospital Universitario, Servicio de Hematología, Salamanca, Spain; ^2^ Centro en Red de Medicina Regenerativa y Terapia Celular de Castilla y León, Salamanca, Spain; ^3^ Centro de Investigación del Cáncer, Universidad de Salamanca, Salamanca, Spain; ^4^ Departamento de Bioquímica y Biología Molecular, Universidad de Salamanca, Salamanca, Spain; ^5^ Unidade de Investigação em Patobiologia Molecular, Instituto Português de Oncologia de Lisboa, Lesboa, Portugal

**Keywords:** HDAC8, bone marrow-mesenchymal stromal cells, myeloproliferative neoplasm cell lines, apoptosis, myeloproliferative neoplasms

## Abstract

Histone deacetylases (HDACs) are involved in epigenetic modulation and their aberrant expression has been demonstrated in myeloproliferative neoplasms (MPN). HDAC8 inhibition has been shown to inhibit JAK2/STAT5 signaling in hematopoietic cells from MPN. Nevertheless, the role of HDAC8 expression in bone marrow-mesenchymal stromal cells (BM-MSC) has not been assessed. In the current work we describe that HDAC8 is significantly over-expressed in MSC from in JAK-2 positive MPN compared to those from healthy-donors (HD-MSC). Using a selective HDAC8 inhibitor (PCI34051), we verified that the subsequent decrease in the protein and mRNA expression of HDAC8 is linked with an increased apoptosis of malignant MSC whereas it has no effects on normal MSC. In addition, HDAC8 inhibition in MPN-MSC also decreased their capacity to maintain neoplastic hematopoiesis, by increasing the apoptosis, cell-cycle arrest and colony formation of JAK2^+^-hematopoietic cells. Mechanistic studies using different MPN cell lines revealed that PCI34051 induced their apoptosis, which is enhanced when were co-cultured with JAK2V617F-MSC, decreased their colony formation and the phosphorylation of STAT3 and STAT5. In summary, we show for the first time that the inhibition of HDAC8 in MSC from JAK2^+^ MPN patients selectively decreases their hematopoietic-supporting ability, suggesting that HDAC8 may be a potential therapeutic target in this setting by acting not only on hematopoietic cells but also on the malignant microenvironment.

## INTRODUCTION

Philadelphia-negative myeloproliferative neoplasms (MPN) comprise a heterogeneous group of malignant clonal hematopoietic diseases, which classically include polycythemia vera (PV), essential thrombocythemia (ET) and primary myelofibrosis (PMF). The JAK2V617F mutation, which results in constitutive activation of the JAK tyrosine kinase and its downstream signaling pathways [1, 2], has been found in most patients with Ph-negative MPN. The constant activation of these signals leads to abnormal proliferation of myeloid cells. It has been demonstrated that the cross-talk between hematopoietic cells and bone marrow (BM) microenvironment influences leukemic stem cell proliferation, survival and migration, contributing to the progression of MPN [3, 4].

Bone marrow mesenchymal stromal cells (BM-MSC) are recognized as the one of the essential elements of both healthy and leukemic hematopoietic microenvironments. Different studies have suggested that leukemic stem cells (LSC) and their progeny secrete high levels of pro-inflammatory cytokines, thus creating a paracrine feedback loop that directly stimulate BM-MSC to overproduce altered osteoblasts as well as inflammatory myelofibrotic cells [5–8].

It has been recently shown the importance of histone deacetylases (HDACs) in myeloproliferation disorders [9, 10]. HDACs constitute a large family of enzymes, which were originally identified as key regulators of nucleosomal histone acetylation controlling gene transcription. They are also involved in other processes, where specific HDAC have been shown to deacetylate non-histone proteins, such as TP53 or α-tubulin (a cytoskeletal protein) [10]. HDAC8 is a class I HDAC involved in various diseases, including cancer [11, 12]. Prior reports have shown the role of this protein in MPN, demonstrating that HDAC8 downregulates the suppressor of cytokine signaling 1/3 (SOCS1/3) expression. HDAC8 knockdown resulted in an increased expression of SOCS1/3, thus leading to a change in JAK2 signal, which translated in a reduced cell growth and clonogenic activity of hematopoietic cells derived from patients with MPN [9, 13].

In previous studies, we and others have shown that MSC from MPN show different gene expression pattern when compared with MSC from normal donors [14, 15]. However, to our knowledge, HDAC8 expression has not been assessed in BM-MSC from MPN patients. In the current manuscript, we have analyzed its expression in BM-MSC from MPN, and studied its potential role in the pathophysiology of these entities.

## RESULTS

### Expression analysis of HDAC8 in BM cells from MPN patients

**Figure 1:**
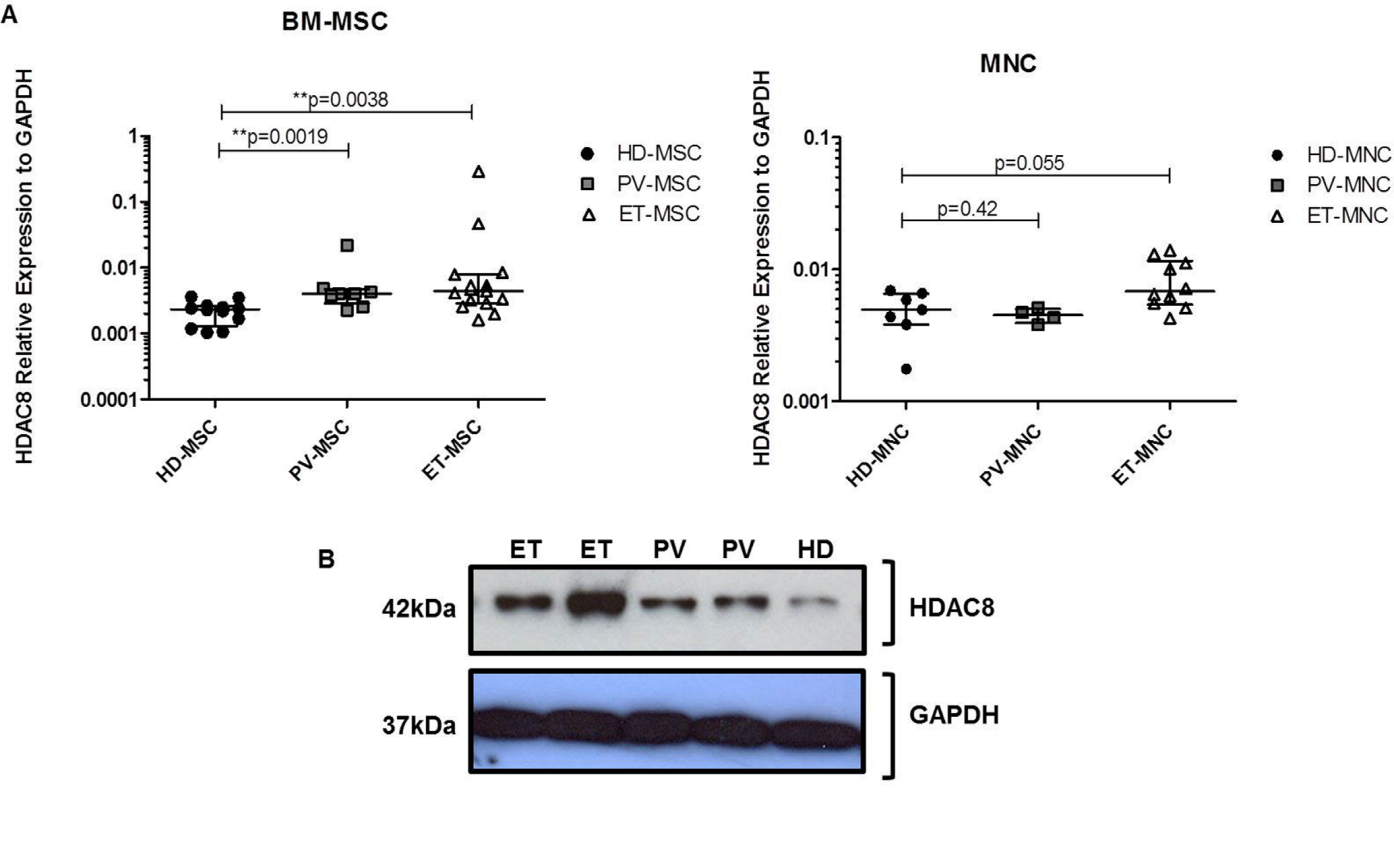
HDAC8 expression (mRNA and protein) (**A**) Expression of HDAC8 gene tested in BM-MSC (left panel) and MNC (right panel) from MPN patients and HD. Results were normalized with GAPDH housekeeping gene. HD-MSC (*n* = 12), PV-MSC (*n* = 8) and ET-MSC (*n* = 15). For MNC, HD = 8, PV = 4 and ET = 10. *p < 0.05 and **p < 0.01. Results are represented as median and range. (**B**) Representative western blot analysis of HDAC8 expression in BM-MSC from three independent experiments performed.

We first compared the mRNA expression of HDAC8 in BM-MSC from JAK2V617F patients (*n* = 8 for PV and *n* = 15 for ET) and HD (*n* = 12). We observed a significantly increase (*p* = 0.0019 for PV and *p* = 0.0038 for ET) of mRNA HDAC8 expression in JAK2V617F-MSC compared to HD-MSC (Figure 1A). We also evaluated the gene expression of HDAC8 in the MNC, that was increased (close to statistical significance; *p* = 0.055) in ET-MNC compared to HD-MNC (Figure 1A). No differences were observed in the mRNA expression of HDAC8 between PV-MNC and HD-MNC. Regarding to HDAC8 protein expression, JAK2V617F-MSC showed an increase in the expression of this protein when compared to HD-MSC, especially in ET-MSC (Figure 1B).

### PCI34051 decreases HDAC8 expression in JAK2V617F-MSC, modifying their cell proliferative capacity

**Figure 2:**
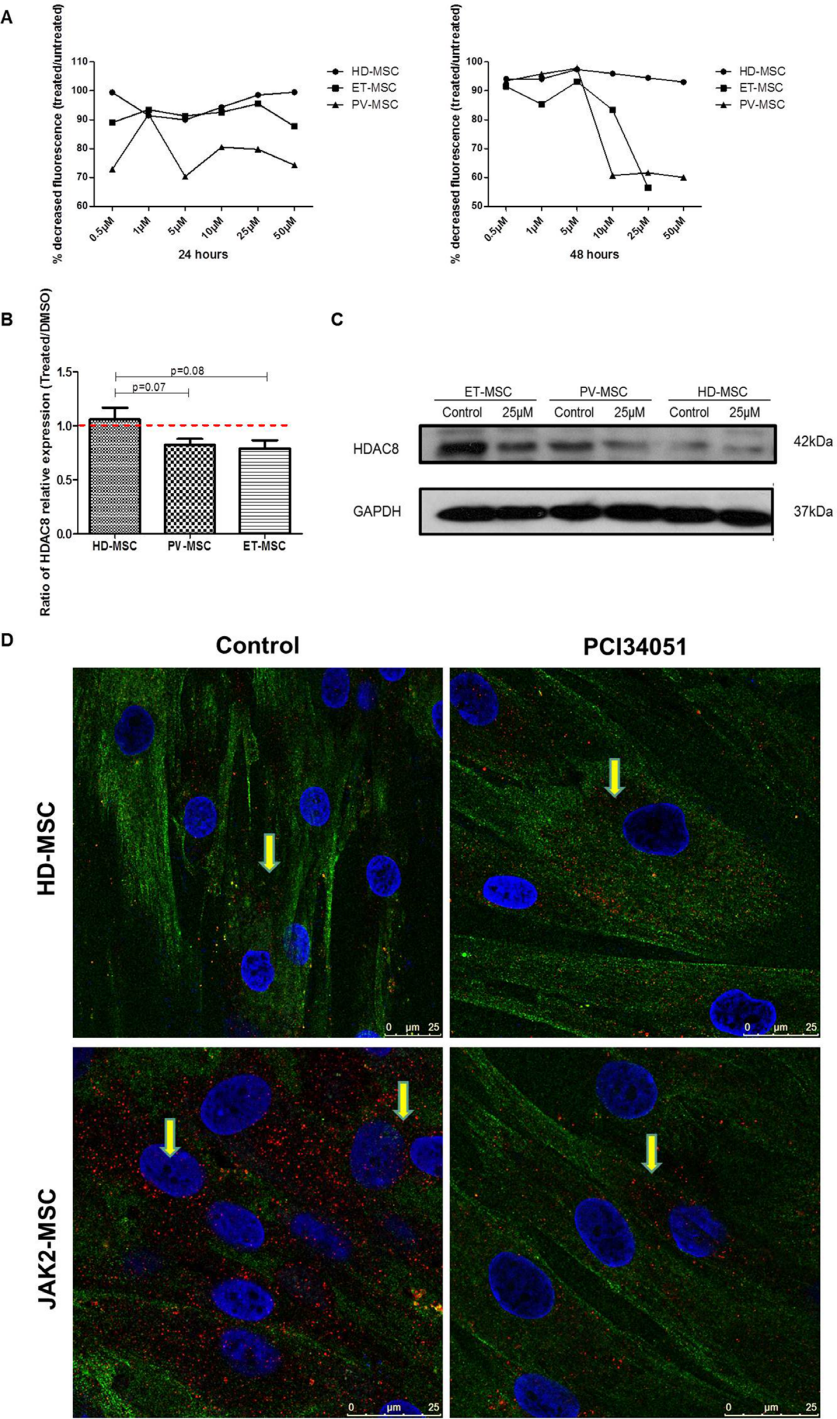
HDAC8i decrease the expression of HDAC8 in BM-MSC from JAK2V617F patients (**A**) PCI34051 induces an AlamarBlue reduction (fluorescence) in BM-MSC from JAK2V617F patients, after treatment for 24 hours and 48 hours. (**B**) Ratio of HDAC8 mRNA expression (Treated cells/untreated), showing that the treatment for 48 h with PCI34051 (25 μM) decreased the expression of HDAC8 in PV and ET-MSC. Data are expressed as mean ± SEM of 3 to 5 independent experiments. (**C**) Decreased expression of HDAC8 in BM-MSC from ET and PV treated with HDAC8i by WB, without changes in HD. (**D**) Representative immunohistochemical images of HD-MSC (upper panel) and MPN-MSC (lower-panel) without treatment (left panel) and after treatment (right panel). Red dots show the localization of HDAC8 in the cells, where can be found mainly in the cytoplasm but also in the nucleus. Green represents tubulin. The scale bar represents 50 and 25 μm.

Because HDAC8 was significantly overexpressed in MPN-MSC we wanted to know whether this molecule could be involved in the functional properties of MSC. For this purpose, the effect of the specific HDAC8 inhibitor (HDAC8i) in BM-MSC cell growth of HD (*n* = 4), ET (*n* = 4) and PV (*n* = 4) was studied. PCI34051 induced a decrease in cell proliferation on the BM-MSC from JAK2V617F patients after 24 hours of treatment. However, at 48 hours of treatment, a wider decrease in cell proliferation in ET and PV-MSC was observed (Figure 2A). HD-MSC maintained their proliferation during the treatment.

Next, we aimed to determine whether HDAC8i could modify the expression of HDAC8 in BM-MSC. As illustrated in Figure 2B, after 48 hours of exposure to 25 μM of PCI34051, the HDAC8 expression ratio between treated and untreated cells was decreased in BM-MSC from JAK2 patients. Regarding protein expression, a decrease in PV and ET-MSC was also observed, with no changes in HD-MSC (Figure 2C and 2D).

**Figure 3:**
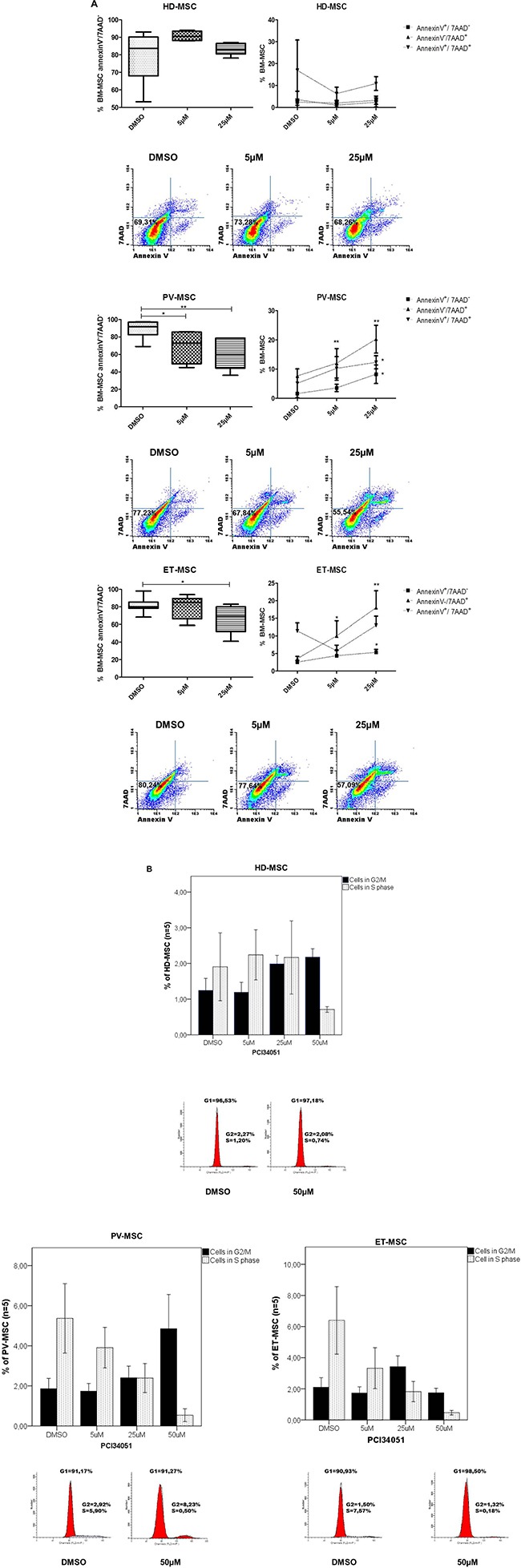
PCI34051 treatment decreases the viability and changes the cell cycle of BM-MSC from JAK2V617F (**A**) Dotplot and Boxplot results showing the viability of BM-MSC by annexinV assay. ET-MSC and PV-MSC showed a significant decrease of viable cells with significant increase of apoptotic cells. * *p* < 0.05 and ** *p* < 0.01. For apoptosis analysis, BM-MSC cells were gated for CD90 (FITC) and CD105 (APC). Then, events were classified as viable cells (annexin-V^neg^/7AAD^neg^), early apoptotic cells (annexin-V^+^/7AAD^neg^), late apoptotic cells (annexin-V^+^/7AAD^+^) and dead cells (annexin-V^neg^/7AAD^+^). (**B**) Cell cycle profiling on BM-MSC after 48 hours of drug incubation. Cell cycle profiling after 48 hours of HDAC8i incubation with different concentrations. The agent induces S-phase reduction when compared to the untreated condition (DMSO). Data are represented as median of 5 experiments for each group. *p < 0.05.

To further investigate the role of HDAC8 inhibition on BM-MSC, its effects on apoptosis and cell cycle was studied by treating BM-MSC with different doses of PCI34051 (5 μM and 25 μM). As illustrated in Figure 3A, when the cells were treated with a high dose (25 μM) of the inhibitor, a significant increase in the percentage of early (Annexin-V^+^/7AAD^−^) and late apoptosis (Annexin-V^+^/7ADD^+^) (*p* = 0.002 and *p* = 0.001, respectively) was observed in ET-MSC when compared to control. Regarding the effect of PCI34051 on PV-MSC, at lower doses (5 μM) it was able to induce a decrease in the percentage of viable PV-MSC (*p* = 0.03), and this effect increased (*p* = 0.008) at higher concentrations of the compound. Treating HD-MSC with PCI34051 did not induced changes in apoptosis. Cell cycle results showed that after 48 hours of treatment with PCI34051, an increase in the percentage of cells in G1/G2 phase together with a decrease in the percentage of cells in S phase could be observed. These results were more prominent in JAK2V617F-MSC, mainly in ET-MSC (*p* = 0.056). Higher concentrations of the inhibitor (50 μM), induced a significant decrease in the percentage of S-phase on PV-MSC (*p* = 0.025) and ET-MSC (*p* = 0.020) (Figure 3B).

### HDAC8i modifies the capacity of JAK2V617F-MSC to maintain the myeloproliferative hematopoiesis

**Figure 4:**
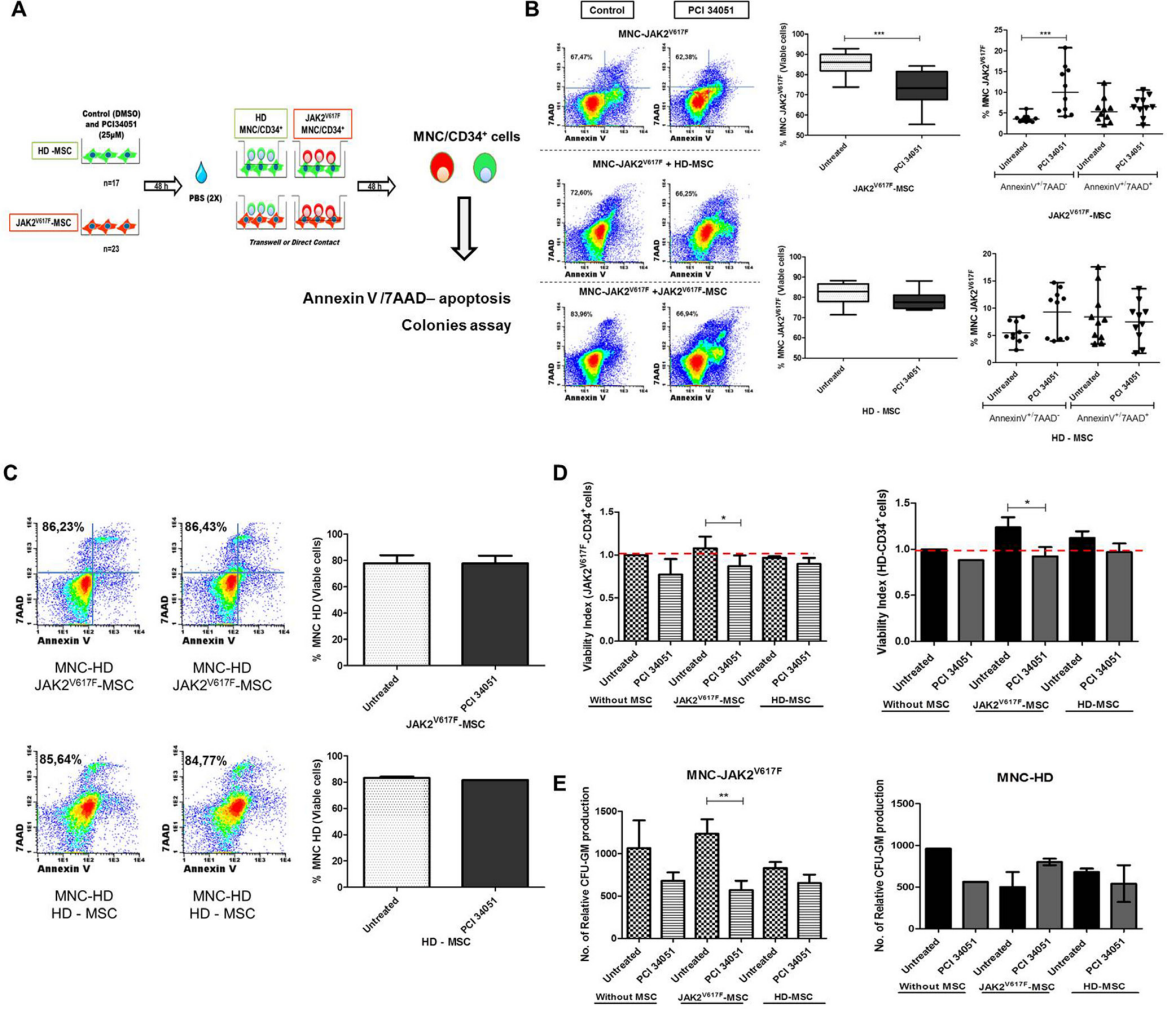
The HDAC8i in the BM-MSC from JAK2 patients decrease the capacity to maintain the neoplastic MNC/HPC (**A**) Scheme of co-culture assays (**B**) Significant increase of early apoptosis in the JAK2V617F-MNC when co-cultured with JAK2V617F-MSC treated with PCI34051 (*n* = 10). No changes when HD-MSC were treated with HDAC8i (*n* = 10) At 48 h of co-culture, MNC/HPC cells were harvested, stained with CD45 and CD105 (to distinguish between hematopoietic cells (CD45+) and the stromal cells (CD105+)) and annexin-V/7AAD to determine cellular viability by flow cytometry analysis. (**C**) HD-MNC were co-cultured with HD and JAK2-MSC with or without treatment (*n* = 4). (**D**) Viability analysis, left column viability of CD34^+^ cells from BM of MPN patients (*n* = 5). Right column represents the BM from HD (*n* = 4). Treatment with PCI34051 in the JAK2V617F-MSC decreases viability of CD34^+^ cells from HD (*n* = 4) and JAK2V617F patients (*n* = 5). The graphs indicate viability index that normalizes the viability values to those of the control conditions (**E**) Relative numbers of CFU-GM from JAK2V617F-MNC and HD-MNC co-cultured with HD (*n* = 6) and JAK2-MSC (*n* = 10) treated with PCI 34051. *p < 0.05.***p < 0.001. Data are represented as median and range.

Because the HDAC8 inhibitor induced changes in MSC from patients we analyzed whether these could translate in changes in their hematopoiesis support capacity. First of all, using co-cultures assays (Figure 4A) we could observe that the inhibition of HDAC8 did not modify the viability of the JAK2V617F-MNC without stroma (Supplementary Figure 1B). However, in the presence of JAK2V617F-MSC previously treated with PCI34051, a significantly decrease the viability of the JAK2V617F-MNC (*p* = 0.0006) was observed, with a significant increase in early apoptosis (*p* = 0.0001) (Figure 4B). The same results were not observed when the JAK2V617F-MNC cells were co-culture with HD-MSC. Figure 4C shows that the treatment of HD-MSC and JAK2V617F-MSC with PCI34051 did not affect healthy MNC viability (*p* = 0.1431).

Since these studies were performed with MNC, we wanted to know if similar results could be also confirmed with CD34^+^ cells. As seen in the Figure 4D, we observed that the inhibition of HDAC8 in JAK2V617F-MSC induced significant decrease of viable CD34^+^ cells (Annexin V^−^/7AAD^−^) from both HD (*p* = 0.028) and JAK2V617F patients (*p* = 0.015). The treatment of HD-MSC had no effect in the viability of CD34^+^ cells, from both HD and JAK2V617F patients. We also evaluated if the observed effects were maintained, when the stroma and hematopoietic cells were co-cultured in direct contact or in the *transwell* assay. MNC from BM of JAK2V617F patients were co-cultured in direct contact (*n* = 19) and in *transwell* - T (*n* = 19) with MSC from HD (*n* = 9) and JAK2V617F (*n* = 10) previously treated with PCI34051, and MNC viability was evaluated. The percentage of viable JAK2-MNC was significantly decreased, when they were cultured with treated JAK2V617F-MSC both in direct contact and *transwell* (Supplementary Figure 2).

In order to see if the effect could also reduce the clonogenic capacity, colony assays were performed. We assessed that HDAC8 inhibition in BM-MSC changed the colony formation ability of MNC when co-cultures were done. As can be observed in Figure 4D the capacity of the myeloproliferative MSC to maintain the malignant MNC was reverted when the MPN-MSC were treated with PCI34051 (*p* = 0.0086). No differences in the clonogenic production was observed when HD-MSC (*n* = 5) were treated with HDAC8i.

### HDAC8i induces apoptosis in myeloproliferative cell lines HEL, SET-2 and UKE-1

**Figure 5:**
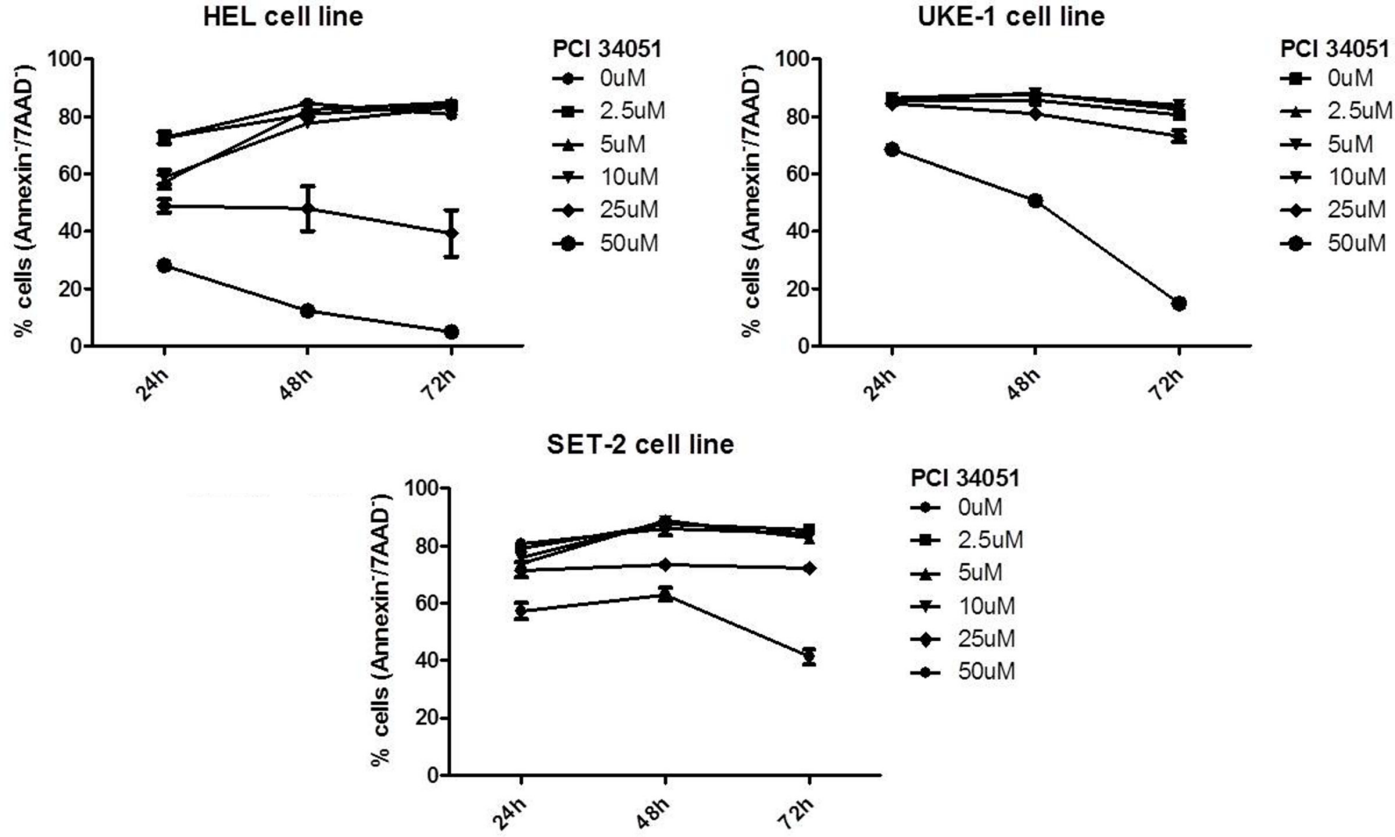
HADC8i in myeloproliferative cell lines (SET-2, UKE-1 and HEL) decreases the cell viability

To determine if HDAC8 inhibition affects the apoptosis of myeloproliferative cell lines, three different cell lines (HEL, UKE-1 and SET-2) were treated for 72 hours with increasing concentrations of inhibitor. As shown in Figure 5, the inhibitor induces cell death at higher concentrations (25 and 50 μM). HEL was the cell line most sensitive to the HDAC8i, so that after only 24 hours of treatment with PCI34051 at concentrations 25 and 50 μM the percentage of viable cells was less than 50%. After 48 and 72 hours of culture with these concentrations of the drug almost all cells were dead. SET-2 and UKE-1 were less sensitive to the inhibitor, although we observed a marked decrease of viable cells 72 hours after treatment at a concentration of 50 μM.

**Figure 6:**
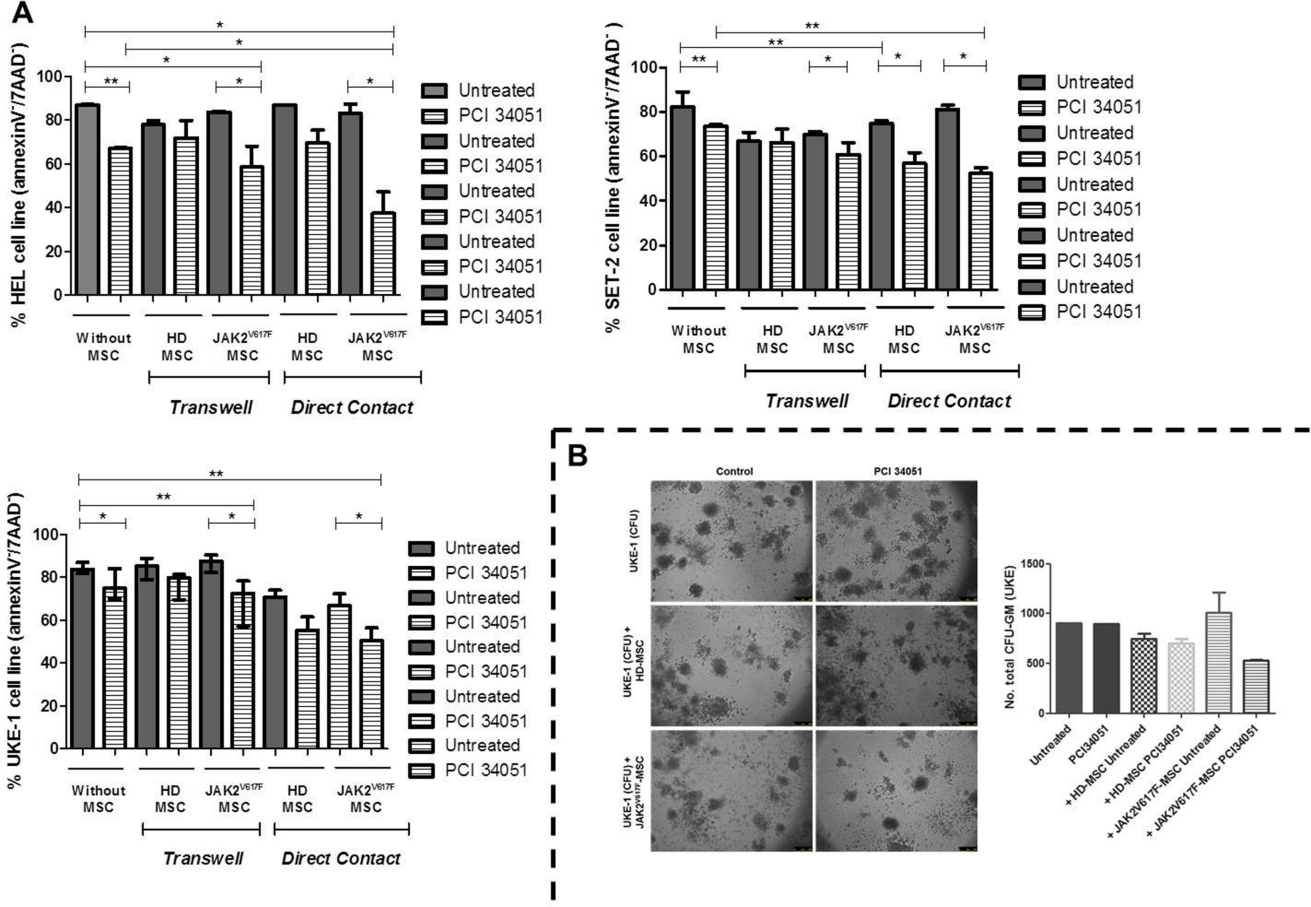
The treatment with PCI34051 induces apoptosis and decreases the colony capacity of MPN cell lines in the presence of MPN-MSC (**A**) HEL, SET-2 and UKE-1 cell lines were cultured *in vitro* (no stroma) and co-cultured with BM-MSC (HD and JAK-2 patients) stromal layer and or separated by a 3 μm-thick micropore membrane (*transwell*). MPN cell lines were incubated in these conditions for 48 h and treated with PCI34051 (25 μM). At 48 h of co-culture, MNP cell lines were harvested, and stained with CD45 and CD90 and annexin-V/7ADD to determine cellular viability. Values indicate the median with the interquartile range of 4 experiments for each condition.*p < 0.05 **p < 0.01. (**B**) CFU-GM assays. No significant differences were observed (*n* = 3).

To evaluate the effects of HDAC8 inhibition on both hematopoietic and stromal cells compartments, we next analyzed if the capacity of PCI34051 to induce cell apoptosis was maintained in the presence of MSC from HD and JAK2V617F patients, addressing this question by co-culture assays. The different cell lines were cultured by *transwell* (3 μm) or in direct contact with BM-MSC, and treated with PCI34051. After 48 h, the hematopoietic cell lines were recovered and the percentage of apoptotic cells was analyzed (Supplementary Figure 3). The presence of BM-MSC from HD and JAK2V617F patients did not change the cell viability of the different cell lines. The three JAK2^+^ cell lines showed a significant decrease of viable cells when treated with PCI34051. However, this decrease was enhanced in the presence of JAK2V617F-MSC, both in direct contact and in *transwell* (Figure 6A). Furthermore, we treated the SET-2 and UKE-1 with HD-MSC and JAK2-MSC for 96 hours with the selective inhibitor, PCI34051, to evaluate if the treatment decreased the cells viability for long periods. We observed a significant decrease (*p* < 0.0001) in the viability of both cell lines, when treated with the compound in the presence of MPN-MSC (Supplementary Figure 4A and 4B). Because we observed these results we performed in a new series of experiments the same methodology, but instead of using MPN cell lines we performed with primary cells. MNC from JAK2V617F patients were co culture with healthy and neoplastic stroma and treated for 96 hours with PCI34051. We observed and high significant decrease in the percentage of viable MPN-MNC cells (Annexin-V^−^/7AAD^−^) when were treated in the presence of JAK2V617F-MSC (Supplementary Figure 4C).

The clonogenic capacity of UKE-1 cells was decreased, without significant differences, when was treated with PCI34051 and in presence of MPN-MSC (Figure 6B).

**Figure 7:**
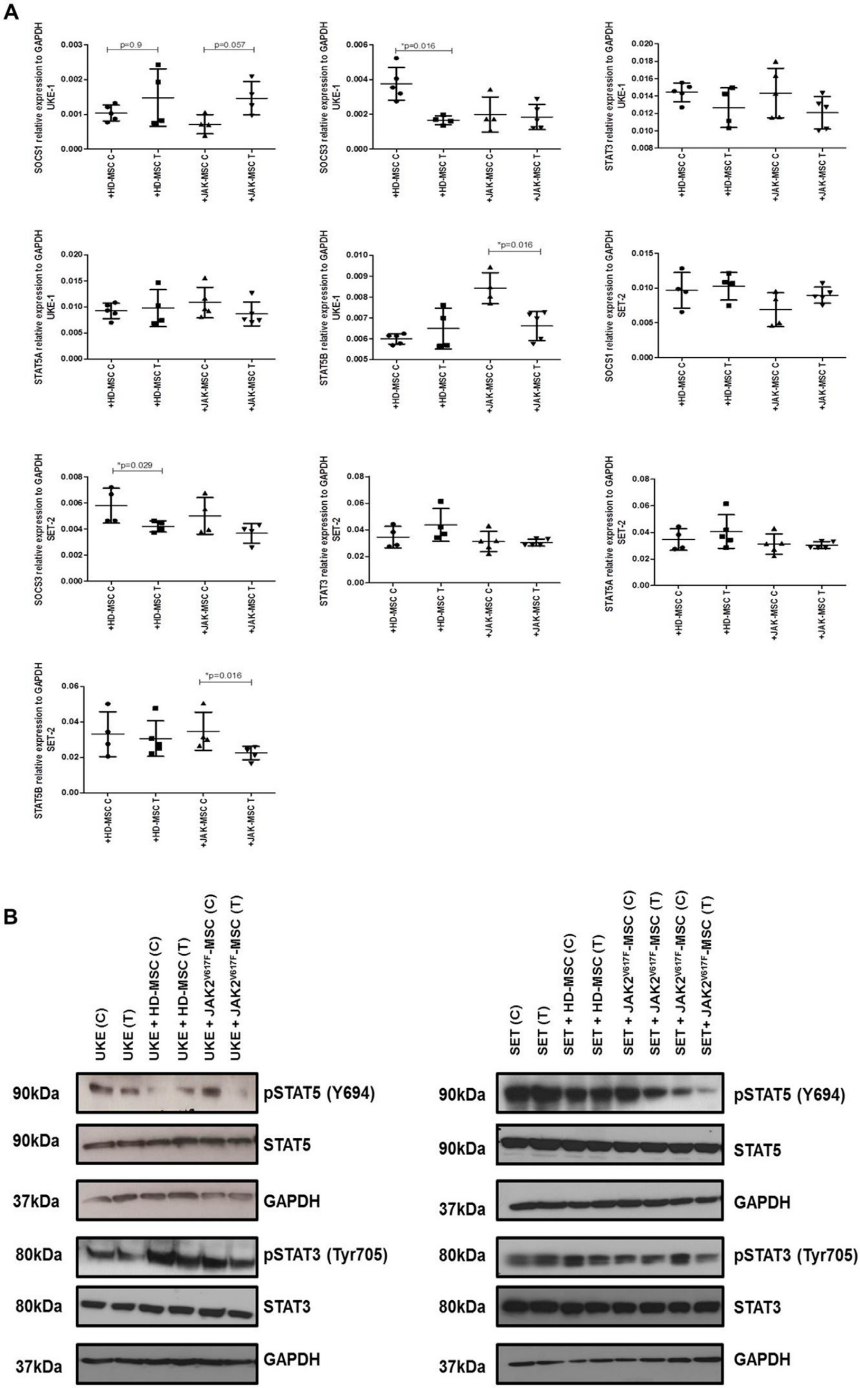
PCI34051changes the expression of crucial signaling pathways in MPN cell lines in presence of neoplastic stroma (**A**) Results are expressed as relative expression compared with untreated cells. Each value is the mean ± SD of four experiments. *p < 0.05. (**B**) Immunoblot analysis. GAPDH was used as loading control. Data are representative of two independent experiments.

To determine the molecular mechanism by which the presence of MPN-MSC induces the apoptosis of MPN cell lines in the presence of PCI34051, we analyzed the changes in activation of various signaling pathways. These included the assessment of the phosphorylation of STAT5 and STAT3 and by RT-PCR of STAT3, STAT5A/B and SOCS1/3. As shown in Figure 7A, a significant decrease (*p* = 0.016) of STAT5B expression when the MPN cell lines (UKE-1 and SET-2) were treated with PCI34051 in the presence of JAK2-MSC.

Thereafter, we studied the phosphorylation of STAT3 and STAT5, where we found a decrease in the phosphorylation of these proteins, mainly in pSTAT5, when both MPN cell lines where treated with HDAC8i in the presence of JAK2V617F-MSC (Figure 7B).

## DISCUSSION

Despite increased knowledge of their molecular pathophysiology, therapeutic strategies for the classic Philadelphia-Negative MPN are currently limited. JAK inhibitors ameliorate symptoms and reduce splenomegaly, but do not reduce the disease burden. Given the limitation of JAK inhibition as monotherapy [16], novel therapeutic strategies are needed.

Post-translational protein modifications such as acetylation or methylation play an important role during epigenetic regulation [17]. Histone deacetylases (HDACs) have gained particular interest as potential targets for novel therapeutic drugs in cancer. HDACs not only are involved in deacetylation of chromatin that can regulate gene-transcription regulation, but also are involved in the deacetylation of non-histone proteins, which regulate cellular homeostasis (cell-cycle, differentiation and apoptosis) [11]. Eighteen mammalian HDACs enzymes have been identified so far, which can be subdivided into two classes, according to their homology.

HDAC activity has been shown to be increased in PMF patients compared to other MPN patients and HD. Results from a small cohort of PMF patients (*n* = 17) has shown aberrant HDAC expression profile in their CD34^+^ cells when compared to controls, with heterogeneous alterations in HDAC subgroups, mainly in HDAC1, 2, 8 and class II HDACs, which are upregulated, whereas HDAC4 and 5 are downregulated [18].

HDAC inhibitors (HADCi) induce a complex cellular response involving both transcriptional and posttranscriptional modulation [12]. Two pan-HDACi are currently under clinical investigations. Panobinostat induces dose-dependent apoptosis of HEL cells (JAK2V617F) by inhibiting the autophosphorylation and expression levels of JAK2V617F, followed by reduced phosphorylation of STAT3, STAT5, ERK1/2 and AKT proteins [19]. The tolerability of this pan-inhibitor in the preclinical studies led to the establishment of a phase Ib clinical trial evaluating the combination of a JAK2 specific inhibitor (TG101209) with panobinostat in MPN patients [20].

The other agent under investigation is the pan-HDACi vorinostat, which also increases apoptosis of MPN cells. Cardoso et al., have recently demonstrated that the combination of vorinostat and ruxolitinib inhibited the differentiation, induced apoptosis and decreased reactive oxygen species production in MPN-cell lines and primary MPN samples [21]. The authors also highlighted that these effects were attenuated in the presence of the HS-5 marrow stromal cells, by the up-regulation of antiapoptotic genes and pro-survival pathways (PI3K-AKT). These results show how bone-marrow niche can protect MPN cells from apoptosis of HDACi or JAK2i [21]. Importantly, the most significant side effects of this pan-HDACi are those related to myelotoxicity [22].

Given the apparently low therapeutic index of HDACis with associated toxicity, and considering our initial observation of the increased expression of HDAC8 characteristically in MSC-MPN JAK2V617F, we decided to further investigate the role that HDAC8 could have in the biology of MSC from MPN patients and later on the effects of HDAC8 inhibition on the behavior of MSC-MPN by using a specific inhibitor PCI34051. HDAC8 is a class I histone deacetylase, which has emerged as an attractive target for drug development in cancer. It has been demonstrated that the overexpression of this protein in multiple tumors, including neuroblastoma, glioma and childhood acute lymphoblastic leukemia, where the use of isoform-specific or selective HDAC8i showed relevant therapeutic results. But, as it is well known the microenvironment is involved in the MPN pathophysiology. We wanted to know whether the effect of this drug could be exerted by acting also on the BM microenvironment.

We found that HDAC8 expression is selectively higher in BM-MSC from MPN-JAK2V617F patients compared to HD-MSC. Furthermore, we studied the impact of the inhibition of HDAC8 in BM-MSC using a selective inhibitor, PCI34051. This compound was demonstrated to induce caspase-dependent apoptosis in cell lines derived from T-cell lymphomas [23]. We observed a decrease in cell proliferation, which was followed by an increase in apoptosis of BM-MSC from JAK2V617F treated samples. Nevertheless, in BM-MSC from HD, treatment with PCI34051 did not induce significant apoptosis. Regarding proliferation, we want to point out that under standard conditions the BM-MSC from MPN-JAK2V617F patients showed similar proliferative capacity compared to BM-MSC from HD (data not provided). These data suggests that the PCI34051 acts specifically in neoplastic BM-MSC, as it would be expected due to the overexpression of HDAC8 in these cells.

Next, we studied the impact of HDAC8 inhibition in one of the main functions of BM-MSC, which is the capacity to support hematopoiesis. After treating BM-MSC from HD and MPN-JAK2V617F with PCI34051, we performed co cultures with MNC and HPC from HD and MPN patients. We observed that MPN-MSC treated with PCI34051 enhanced the apoptosis of JAK2V617F-MNC, and decreased their clonogenic capacity. Then, we assessed the effects of HDAC8 inhibition in JAK2V617F-MSC when co-cultured with HPC. Similarly, we observed a decrease in MPN-CD34^+^ cell viability and a decrease in CFU-GM formation. By contrast, no significant changes in HD-HPC viability and CFU-GM formation were observed, after being in co-cultured with treated BM-MSC. We then planned to study if the previously described effects were maintained, when MPN-cell lines (hematopoietic component) and MPN-MSC were treated with HDAC8i simultaneously. A decrease in the viability of HEL, SET-2 and UKE-1 (all JAK2 cell lines) was shown when treated with PCI34051, which was increased when co-cultured with MPN-MSC. An increase in the apoptosis of these MPN cell lines was associated with a decrease in colony-formation.

JAK2V617F mutation in hematopoietic stem and progenitor cells confers hypersensitivity to cytokines, which leads to a constitutive JAK2 tyrosine kinase signaling. These changes activate downstream pathways, in particular the phosphorylation of transcription factors STAT3 and STAT5 [24]. Previous studies have demonstrated that STAT5 loss prevents the development of JAK2V617F-induced MPN and is dispensable in normal hematopoiesis [25]. In the present study we have shown that, besides to clonogenic inhibition a decrease in pSTAT5 and pSTAT3 were also observed.

Different reports have been demonstrated how BM-MSC play an important role in the BM hematopoietic niche and the impact of dysfunctional BM-MSC have in the pathogenesis of myeloid malignances [26]. Some studies suggested that MSC can act as drug delivery system. In a recent report it has been shown the capacity of a brief exposure to immunosuppressive drugs to improve the immunoregulatory potency of MSC [27]. In our model, the results suggest that BM-MSC from MPN patients with JAK2V617F mutation does not act as a carrier of the inhibitor but as a specific target for PCI34051. We showed that BM-MSC from MPN patients could not protect the leukemic cells when were treated with HDAC8i by decreasing the activation of the JAK-STAT signaling pathway in the hematopoietic cells. It can be hypothesized that the activation of this pathway seems to be involved in this protective machinery. However, the mechanisms are not completely understood and need to be elucidated.

In summary, MSC from MPN have higher expression of HDAC8 than normal ones, and the inhibition of HDAC8 expression by its specific inhibitor decreases the capacity of the stroma to support hematopoietic cells from MPN patients, suggesting that HDAC8 may be a potential therapeutic target in this setting.

## MATERIALS AND METHODS

### Samples and ethical statements

Bone marrow (BM) aspirates from 17 healthy donors (HD) and 23 newly diagnosed Ph-negative MPN harboring the JAK2V617F mutation were used for BM-MSC isolation and expansion or for mononuclear cell (MNC)/CD34^+^ cells isolation.

Median age of JAK2V617F MPN patients was 62 (range 31–74). Median age of control samples (HD-BM) was 44 (range 21–67), 13 male and 4 female. MPN patient's characteristics and information (diagnosis, gender, age, hematimetric parameter and the percentage of JAK2V617F mutation) are summarized in Table 1. The patients diagnosis was established according to the WHO classification [28]. All the experiments were conducted in accordance to ethical standards and principles expressed in the Declaration of Helsinki, and all the samples were collected after informed consent was obtained. The study was approved by the local Ethics Committee of the Hospital Universitario de Salamanca (Spain).

**Table 1:** Clinical characterisitics of MPN patients

Subject	Gender	Age (y)	Hb (g/dL)	Platelets 10^3^/μL	WBC 10^3^/μL	%JAK2V617F
**PV1**	F	46	16.9	775	11.1	17%
**PV2**	M	53	13.9	262	4.01	49%
**PV3**	F	52	17.3	649	8.11	33%
**PV4**	F	72	16.4	454	14.13	90%
**PV5**	M	66	16.9	1041	17.6	46%
**PV6**	M	67	17.3	485	12.9	33%
**PV7**	F	69	16.7	356	5.87	33%
**PV8**	M	71	18.4	597	8.26	23%
**ET1**	F	40	14.3	415	9.2	24%
**ET2**	F	51	14.1	596	9.17	16%
**ET3**	M	69	14.9	293	6.79	32%
**ET4**	F	51	13.5	644	7.41	13%
**ET5**	M	72	11.8	472	7.98	48%
**ET6**	M	73	12.3	1167	13.7	25%
**ET7**	F	45	15.5	648	10.5	12%
**ET8**	F	56	16.1	856	8.5	20%
**ET9**	M	62	17	697	10.2	10%
**ET10**	M	31	16.6	168	3.78	30%
**ET11**	F	68	14.7	571	6.92	18%
**ET12**	M	74	15.8	387	11.6	16%
**ET13**	F	67	15.8	1027	11.1	24%
**ET14**	F	50	15.3	517	7.78	15%
**ET15**	M	47	14	634	5.22	44%

Abbreviations: F- Female, M- Male, PV - Polycythemia Vera, ET- Essential thrombocythemia, Hb - Hemoglobin, WBC- withe blood cells. %JAK2V617F - represents the percentage of hematopoietic cells with the mutation in the bone marrow or in peripheral blood.

### Cell lines

UKE-1 and HEL cell line (homozygous for JAK2V617F mutation) and SET-2 (heterozygous for JAK2) were used [21]. SET-2 and HEL cell lines were cultured in RPMI (Gibco, Paisley, UK), and UKE-1 in IMDM medium (Lonza, Belgium) supplemented with 10% fetal bovine serum (FBS), 100 U/mL penicillin, 100 μg/mL streptomycin and 2 mM L-glutamine (Gibco, Invitrogen™). All of them were cultured at 37°C in a humidified atmosphere with 5% CO_2_.

### Isolation, expansion and characterization of BM-MSC from MPN patients and HD

Bone marrow mononuclear cells (BM-MNC) were isolated from BM aspirates, separated by density gradient centrifugation with Ficoll-Paque (density: 1.077k GE Healthcare BioSciences) and cultured in standard culture medium as previously described [29]. Isolated BM-MSC were characterized to fulfill the minimal criteria established by the International Society for Cellular Therapy (ISCT) [30]. This includes the capacity to differentiate into osteoblasts and adipocytes, and standard immunophenotypical assessment, as previously described [31]. Cells were cultured at 37°C in a humidified atmosphere with 5% of CO_2_. For all the experiments was used BM-MSC at passage 3–4.

### RNA isolation and gene expression analysis

Total RNA was extracted from BM-MSC and BM-MNC by Trizol (Invitrogen) as previously reported [32]. Relative quantification was calculated using the 2^−ΔCt^ values where: ΔCt = Ct_Gene_–Ct_GAPDH_. The different primers are indicated in Supplementary Table 1. GAPDH housekeeping gene was used for normalization.

### Apoptosis and cell cycle assays

For cellular viability assessment, the BM-MSC cells were washed with PBS twice, and then were trypsinized. The harvest cells were stained with CD90-FITC (BD) and CD105-APC (R&D system). In the case of co-culture assays the harvested MNC and CD34+ cells were stained with CD45-FITC (BD) and CD105-APC. In the case of MPN cell lines assays the harvested cells were previously labeled with CD90-FITC and CD45-APC, and then performed the annexin-V kit, as described below.

Cells from the different experimental conditions were stained with Annexin V-PE using the PE annexin V apoptosis detection kit (BD) following manufacturer's instructions. For cell cycle, the cells were stained with propidium iodite (PI), using the kit cycle tests (BD), according to manufacturer's recommendations. Samples were acquired on a FACSCalibur flow cytometer (BD) and analyzed using the Infinicyt software (Cytognos, Salamanca, Spain). Cell-cycle was analyzed using the ModFit LTTM Macintosh program (Verity Software, USA).

### Immunobloting

Protein extracts and immunoblotting were performed as previously reported [33]. Primary antibodies used were HDAC8 (Abcam#ab187139), pSTAT_3(Tyr705)_ (Cell Signaling), pSTAT5_(Y694)_ (BD), STAT3 and STAT5 (Santa Cruz) and GAPDH (Cell Signaling). The immune reactive protein bands on the membranes were visualized using enhanced chemiluminescence ECL-Plus reagent (Thermo Fischer Scientific, United States of America).

### Immunofluorescence

For immunofluorescence experiments, BM-MSC were fixed with 4% paraformaldehyde and further processed as previously described [34]. Slides were mounted using Vectashield H-1000 medium (Vector Labs) and examined images were captured using a TCS SP5 Confocal Laser Scanning Microscope (Leica Microsystems, Wetzlar, Germany) with the LAS AF acquisition program (version 2.6.0.7266). Fluorescence images were captured using a Leica DMI6000B microscope (Leica Microsystems). Captured images were handled using Adobe Photoshop CS6 (Adobe Systems, San Jose, CA, USA).

### Isolation of hematopoietic progenitor cells

After density gradient separation CD34^+^ cells were selected positively from BM-MNC by immunomagnetic cell separation (Miltenyi Biotec, Bergisch-Gladbach, Germany) as previously published [35].

### BM-MSC HDAC8 inhibition (*in vitro* assays)

To assess the effects of HDAC8 inhibition on the proliferation/cell growth of normal and malignant BM-MSC, a pharmacological selective inhibitor PCI34051 was used. Cells were seeded in 96-well or 24-well plates at a concentration of 3 × 10^3^ cells/well or 10^4^ cells/well, respectively. Then the cells were treated with PCI34051 at different concentrations (0.5 μM, 1 μM, 2 μM, 5 μM, 10 μM, 25 μM, and 50 μM) for 24 h and 48 h. After that, proliferation was measured using Alamar blue as reported previously [36]. After treatment, BM-MSC were incubated for 4–6 hours at 37°C with a solution prepared with AlamarBlue and fresh culture medium (with reduced serum) in a proportion 1:9 (v/v). Fluorescence was measured using a fluorescence excitation wavelength of 540–570 nm (peak excitation is at 570 nm) with a photometric microplane reader in an Infinite^®^ F500 teach plate reader (Tecan; Maennedorf, Switzerland). AlamarBlue activity was calculated subtracting the blank value (well with medium without cells) and normalized with the control (untreated cells).

### Co-culture system

BM-MSC at passage 3 from different individual HD and MPN patients were seeded at concentration of 10^5^ cells/ well in a 6-well plate (Costar, Bodenheim, Germany) overnight. Then BM-MSC were incubated with PCI34051 at concentration of 25 μM for 48 hours. Next, the stromal layers were washed twice with PBS and freshly isolated CD34^+^/MNC cells from BM of HD and JAK2V617F patients were seeded either directly (cell-on-cell) or indirectly (separated by 3.0 μm-thick micropore membranes, Corning Incorporated, Costar).

The MNC:MSC ratio was 10:1 and CD34^+^:MSC was 2:1, according to previous experiments [37, 38]. The cultures were maintained in a 5% CO2, humidified atmosphere at 37°C for 48 hours. Then, hematopoietic cells were recovered to evaluate apoptosis, and to perform RT-PCR and clonogenic assays.

In additional experiments, stromal cells were cultured until reaching 70% of confluence, and then MPN cell lines (UKE-1, SET-2 and HEL) were added to the stromal layer at 2 × 10^5^ cells/mL in culture medium, either in direct contact or separated. After 48 h of treatment with the specific HDAC8 inhibitor, the cell lines were harvested and assessed as described below for apoptosis, colony assay and immunoblotting.

### Clonogenic assays

MNC, CD34^+^ cells and UKE-1 cell lines were recovered after the different *in vitro* system and plated in CFU assays (MethoCult H4534-Stem Cell Technologies, Vancouver, Canada) according to the manufacturer's instructions. After 14 days, CFU were enumerated and classified by morphology as previously described [39].

### Statistical analysis

Statistical analysis was performed using IBM SPSS Statistics 21 (Chicago, IL, USA) and GraphPad Prims version 5.00 for Windows (GraphPad Software). The values reported in the figures are given as median with the interquartile range or with mean ± standard error of the mean (SEM). Differences between populations were calculated using the Mann-Whitney tests with Bonferroni corrections. A *p*-value < 0.05 was considered to be statistically significant.

## SUPPLEMENTARY MATERIALS FIGURES AND TABLES


