# Supplementary material for: HDAC8 overexpression in mesenchymal stromal cells from JAK2+ myeloproliferative neoplasms: a new therapeutic target?
Source: Oncotarget. 2017 Mar 7;8(17):28187–202. doi: 10.18632/oncotarget.15969 (PMC5438642; doi:10.18632/oncotarget.15969)
Supplement: Supplementary file 1 [file oncotarget-08-28187-s001.pdf]

HDAC8 overexpression in mesenchymal stromal cells from $JAK2^{+}$ myeloproliferative neoplasms: a new therapeutic target?

Supplementary Materials

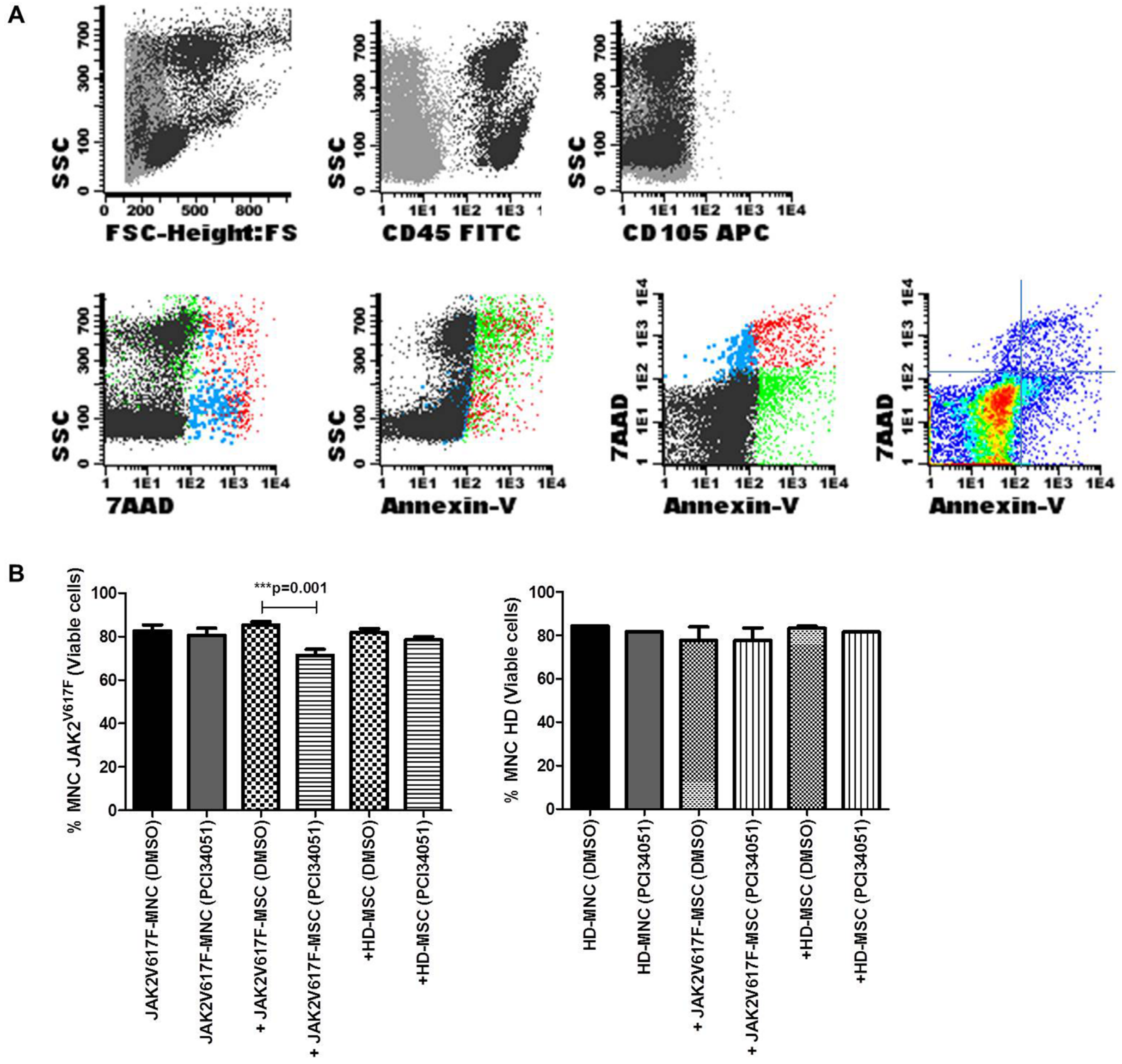

Supplementary Figure 1: (**A**) Representative dotplots images of MNC (HD) treated for 48 hours with DMSO. We selected the cells CD45 (positive for hematopoietic cells) and at the same time negative for CD105 (mesenchymal marker). Then we selected the cells that were both positive for Annexin V and 7AAD (late apoptosis), then the cells that were only positive for annexin V (early apoptosis) and finally the dead cells (positive for 7AAD and negative for AnnexinV). (**B**) Graph bars of the percentage of MNC viable cells ($Annexin^{-}$/$7AAD^{-}$) from HD and JAK2V617F patients without stroma and with stroma (HD and JAK2V617F) previously treated with PCI34051.

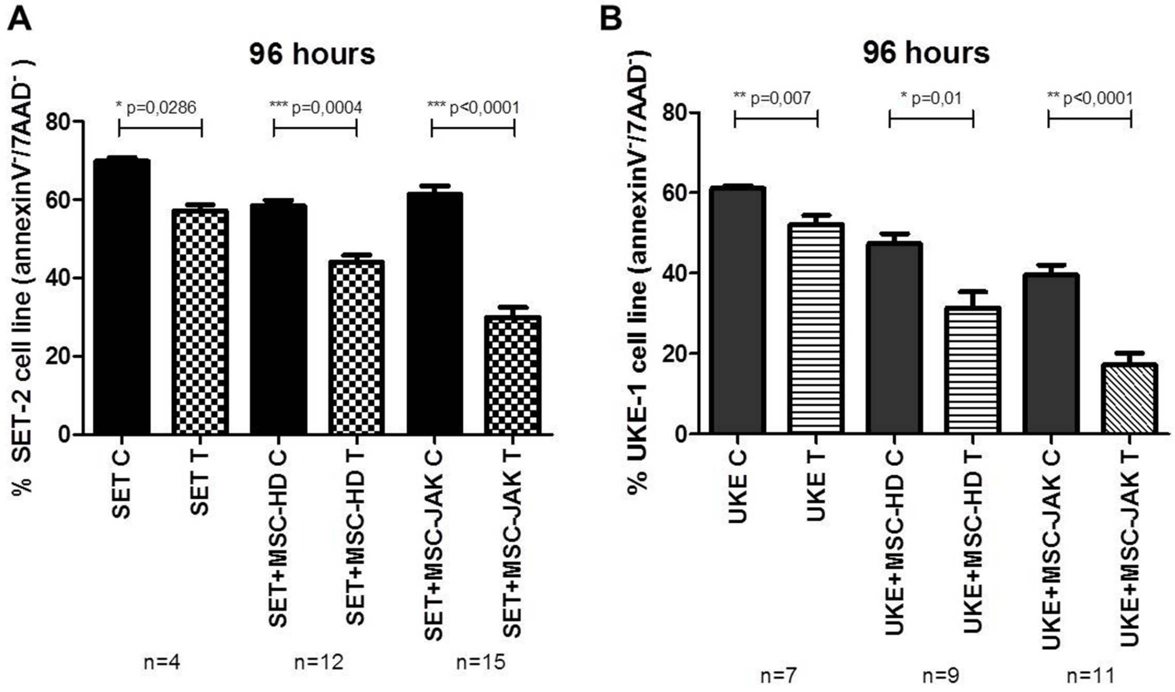

Supplementary Figure 2: HDAC8i in JAK2V617F-MSC increase the apoptosis of pathologic MNC in co-culture systems (direct contact and *transwell*). A significant decrease of JAK2-MNC alive was observed when were co-cultured with JAK2-MSC previously treated with PCI34051 in direct contact (n = 9) or by *transwell* (m = 10). Was not observed differences when the pathologic MNC were co-cultured with HD-MSC treated with PCI34051 in direct contact (n = 9) or in *transwell* (n = 10) *$p < 0.05$. Data are represented as median and range.

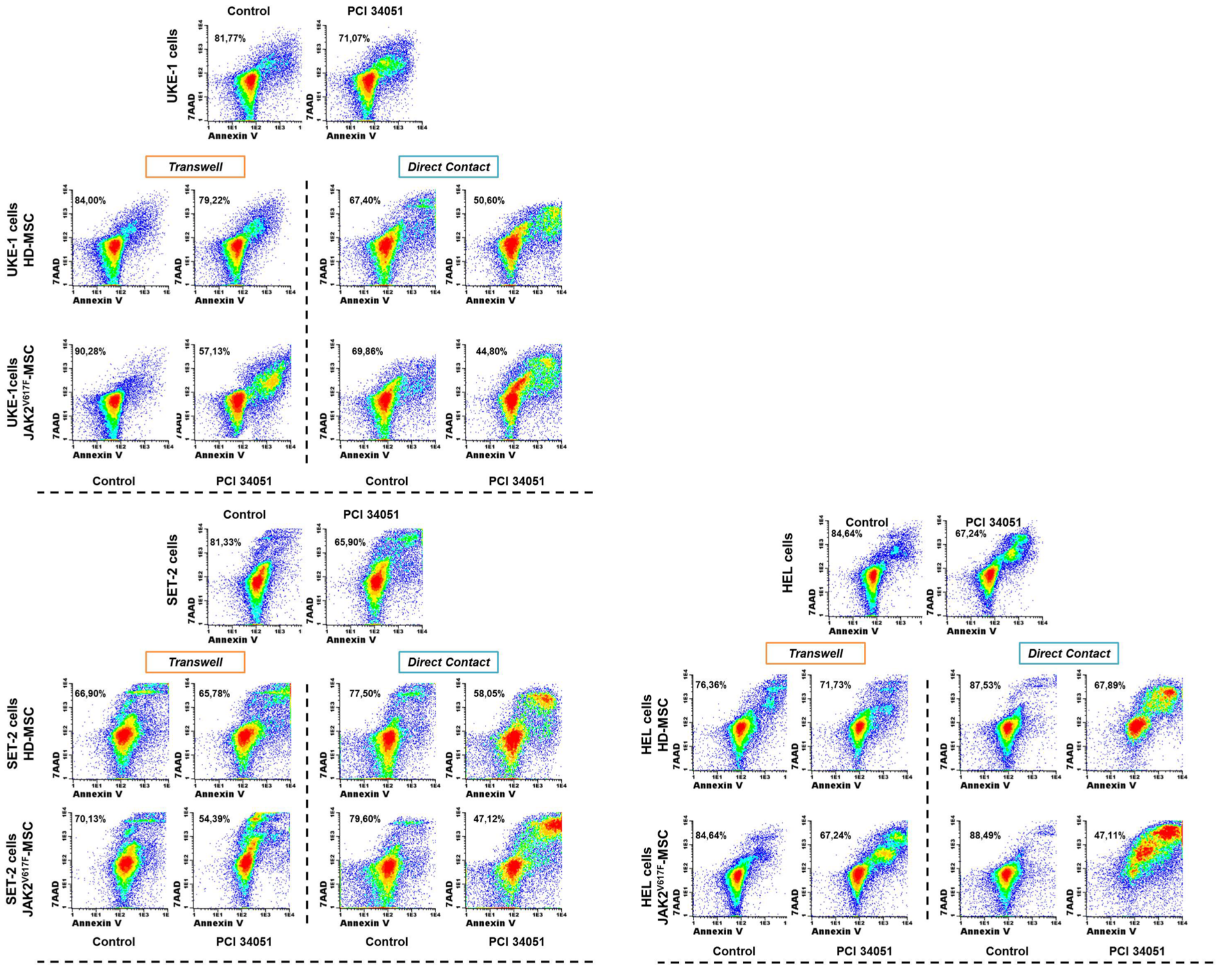

Supplementary Figure 3: Flow Cytometry dot plot images. Apoptosis of the different cell lines (UKE-1, SET-2 and HEL) that were co-cultured with stroma (HD and JAK2V617F) and treated with PCI34051 for 48 h.

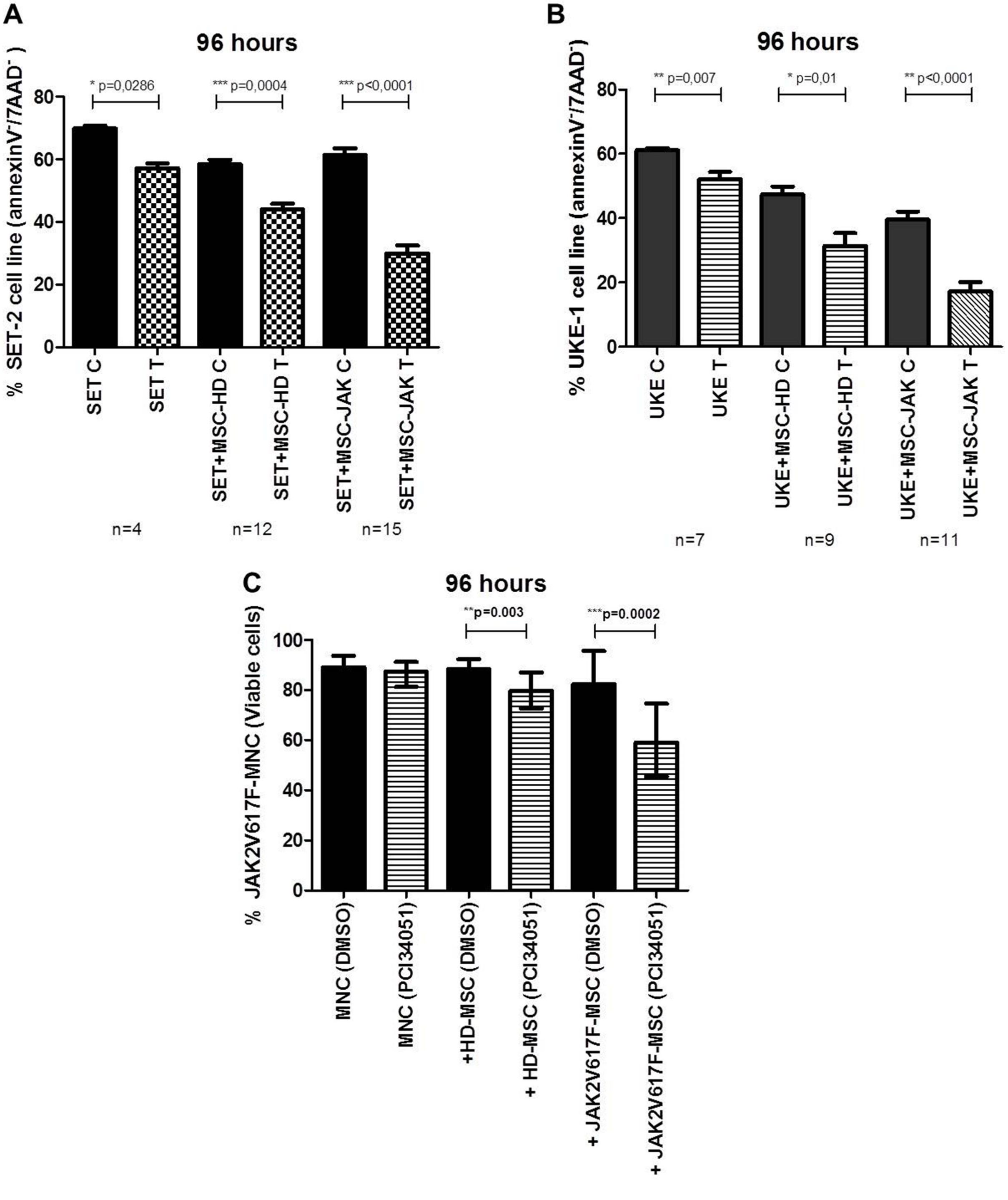

Supplementary Figure 4: The treatment with PCI34051 induces apoptosis of MPN cell lines in the presence of MPN-MSC. (**A**) SET-2 and (**B**) UKE-1 cell lines were cultured with or without BM-MSC (HD and JAK-2 patients) by direct contact for 96 h. (**C**) The results are representative of the percentage of viable MNC cells (Annexin-V and 7AAD negative cells) from JAKV617F patients that were treated with PCI34051 for 96 h without or with HD-MSC (n = 10) and JAKV617F-MSC (n = 12). Values indicate the mean ± standard deviation. (*p < 0.05 ** p < 0.01 ***p < 0.0001).

Supplementary Table 1: Panel of genes used in RT-PCR assays

Gene symbol	Assay IDs
HDAC8	HS00954353
GAPDH	Hs02758991
SOCS3	Hs02330328
SOCS1	Hs00705164
STAT3	Hs00374280
STAT5A	Hs00559637
STAT5B	Hs00560026

All the genes were purchased from Applied Biosystems, Foster City, CA, USA.
